# Bovine leukemia virus: an emerging concern for zoonotic cross-species transmission

**DOI:** 10.3389/fcimb.2025.1720247

**Published:** 2026-01-29

**Authors:** Changqing Yu, Yujing Li, Haojie Wang, Faming Jiang, Chao Xu, Nan Li, Xiaoyang Huang, Bin Zhang, Yang Wu

**Affiliations:** 1 Engineering Center of Agricultural Biosafety Assessment and Biotechnology, School of Advanced Agricultural Sciences, Yibin Vocational and Technical College, Yibin, China; 2 State Key Laboratory for Animal Disease Control and Prevention, Harbin Veterinary Research Institute, Chinese Academy of Agricultural Sciences, Harbin, China; 3 Bamboo Diseases and Pest Control and Resources Development Key Laboratory of Sichuan Province, Leshan Normal University, Leshan, China; 4 Department of Oceanography, Key Laboratory for Coastal Ocean Variation and Disaster Prediction, College of Ocean and Meteorology, Guangdong Ocean University, Zhanjiang, China; 5 College of Animal and Veterinary Sciences, Southwest Minzu University, Chengdu, China

**Keywords:** bovine leukemia virus, breast cancer, cattle, public health, zoonosis

## Abstract

Bovine leukemia virus (BLV) is a retrovirus that causes leukemia-like disorders in cattle and sheep, as well as inflammatory conditions and reduced milk production in dairy cows, leading to substantial economic losses in the global cattle industry. BLV infection is widely prevalent among cattle populations worldwide. Emerging evidence has indicated a potential association between BLV infection and human breast cancer development. In several regions, BLV DNA has been detected in human breast tissue samples, particularly in tumor cells, suggesting that BLV may contribute to the development of breast cancer. This review summarizes recent advances in research on the possible link between BLV and human breast cancer, emphasizing its potential public health significance. Furthermore, we examine progress in the development of preventive vaccines, which may play a crucial role in mitigating BLV transmission.

## 1 Introduction

BLV is a retrovirus capable of infecting various ruminant species, including cattle, yellow cattle, and yaks, leading to enzootic bovine leukosis (EBL). Under experimental conditions, BLV has been shown to infect non-ruminant animals such as goats, sheep, pigs, rabbits, rats, and chickens (Schwartz and Lévy, 1994). Approximately 70% of BLV-infected cattle remain asymptomatic, while 30% develop persistent lymphocytosis (PL), among which only about 5% progress to malignant lymphocyte proliferation after 5–7 years of infection, typically characterized by CD5+ B-cell malignancy (Barez et al., 2015). In addition to elevated lymphocyte counts, BLV infection is associated with reduced milk production (Da et al., 1993), progressive weight loss, immune suppression, and cellular inflammatory responses in infected animals. BLV infections have been reported globally across Asia, Africa, the Americas, and Europe (Marawan et al., 2021). Notably, emerging evidence indicates that BLV may have zoonotic potential and is significantly associated with the incidence of human breast cancer (Buehring et al., 2014). This article reviews current findings on the association between BLV infection and human breast cancer, discusses the mechanisms underlying cross-species transmission, and examines recent advances in the development of preventive vaccines.

## 2 Composition of the BLV genome and its cellular infection cycle

**Figure 1:**
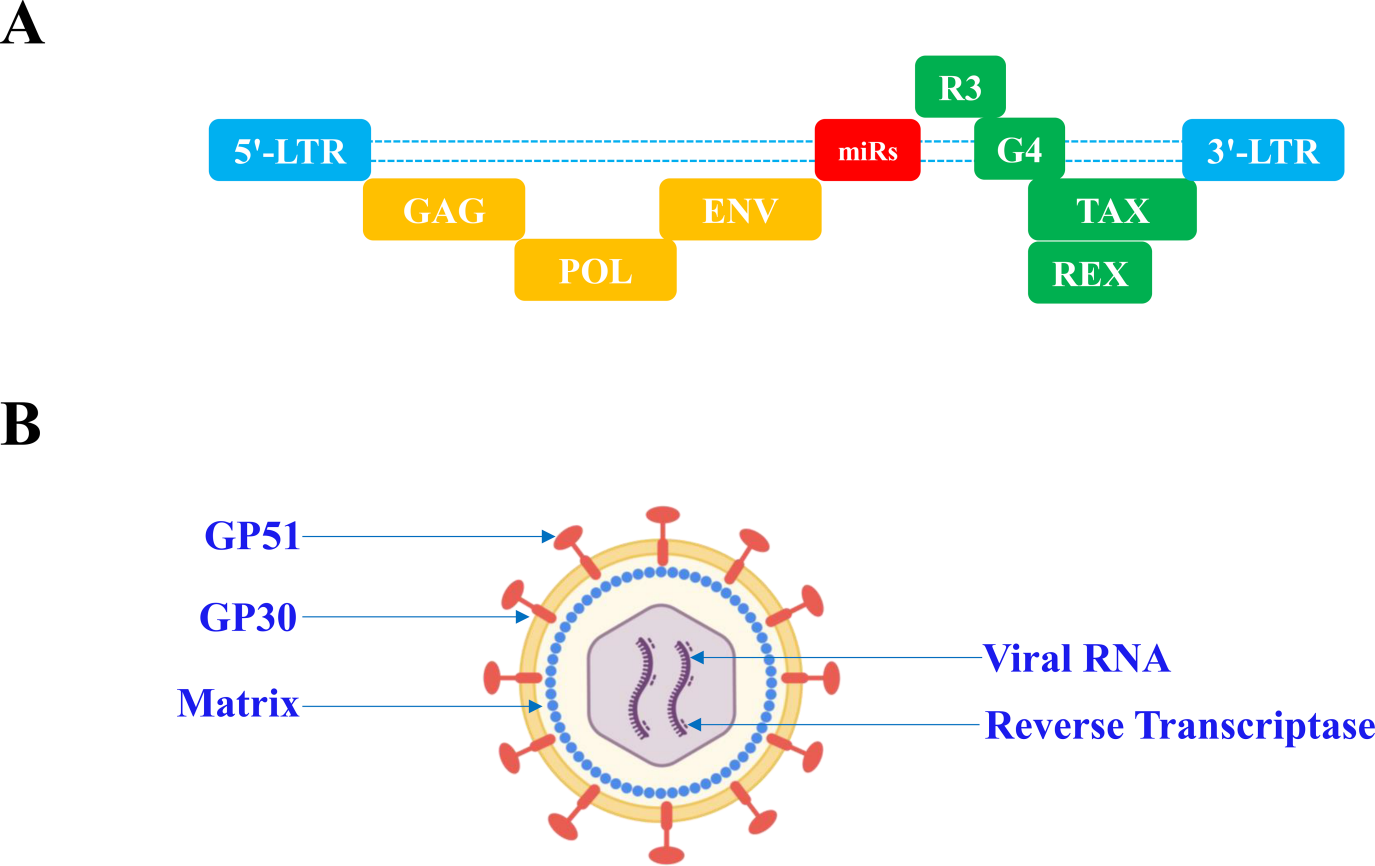
Genetic composition of BLV. **(A)** Genome organization of BLV. **(B)** Major components of BLV virions.

Bovine leukemia virus (BLV) belongs to the *Deltaretrovirus* genus within the *Retroviridae* family and contains long terminal repeat (LTR) sequences at both ends of its genome, designated as 5′-LTR and 3′-LTR. The viral genome encodes four structural proteins, Gag, Pro, Pol, and Env, four regulatory proteins, R3, G4, Rex, and Ta, and a non-coding microRNA (miRNA) region that plays a critical regulatory role during intracellular infection (Lairmore, 2014) (**Figures 1A, B**).

Among these, Gag and Env are key structural proteins and represent potential targets for diagnostic assays and vaccine development. Gag is responsible for packaging viral genomic RNA into nascent virions, while the Env glycoprotein (gp51/gp30) mediates attachment to cellular receptors and facilitates fusion between the viral envelope and host cell membrane. The Tax protein disrupts cellular DNA repair mechanisms, contributing to genomic instability and promoting oncogenesis (Philpott and Buehring, 1999). The intracellular infection cycle of BLV is typical of retroviruses. Following attachment to the host cell receptor cationic amino acid transporter 1 (CAT-1), BLV enters the cell and releases its core components into the cytoplasm (Bai et al., 2019). After uncoating of the viral core, the genomic RNA is reverse transcribed into complementary DNA (cDNA) by reverse transcriptase. This cDNA is then transported into the nucleus as part of the viral pre-integration complex. Once in the nucleus, the cDNA integrates into the host genomic DNA through the action of the viral integrase enzyme. Subsequently, host cell transcription machinery drives expression of viral genes, leading to the synthesis of viral proteins and genomic RNA in the cytoplasm. Newly assembled virions bud from the plasma membrane, completing the viral replication cycle (**Figure 1B**).2.

## 3 The origin and current status of BLV epidemics

Bovine leukemia virus (BLV) was first identified in Lithuania, located along the southeastern coast of the Baltic Sea, in 1871. Since then, BLV infection has been reported in numerous countries worldwide. The United States exhibits the highest prevalence of BLV infection, while Japan, Canada, Argentina, and Mexico also report high infection rates (Marawan et al., 2021). Yang Y et al. conducted a nationwide survey on bovine leukemia across 15 provinces in China and found that BLV infection is prevalent in clinical settings, posing a significant threat to the country’s cattle and dairy industries (Yang et al., 2016). Genotype 6 has been identified as the predominant strain in China (Yang et al., 2022). To date, at least 11 distinct BLV genotypes have been identified based on genetic variations in the Env gene sequence (Yu et al., 2019). However, the comparative virulence among these genotypes remains unclear and requires further experimental investigation.

## 4 Zoonotic potential of BLV

### 4.1 Discovery of BLV in human breast tissue

Bovine leukemia virus (BLV) infection is highly prevalent in cattle herds across the United States. On large dairy farms, infection rates range from as low as 40% to as high as 100% (Buehring et al., 2014). BLV-infected lymphocytes have been detected in blood and milk products from infected cows, suggesting a potential route of human exposure to the virus. Initially, it was believed that BLV could not infect humans (Burridge, 1981). However, Buehring et al. were the first to demonstrate that BLV can infect human breast tissue cells. Their findings revealed that the BLV p24 protein was detected in 12 out of 215 breast tissue samples, some of which were derived from cancerous tissues (Buehring et al., 2014). Subsequent PCR amplification confirmed the presence of BLV proviral DNA in these samples. Based on these findings,

Buehring GC conducted a clinical investigation into the potential association between BLV infection and human breast cancer genesis. Following this work, researchers in other countries have carried out similar studies to further explore this possible link.

### 4.2 BLV infection investigation in the American population

In 2003, Buehring et al. conducted a serological survey to detect bovine leukemia virus (BLV) antibodies in human serum samples, and their results showed that 39% of the tested samples were positive for BLV IgG antibodies (Buehring et al., 2003), indicating prior exposure or ongoing viral replication in humans. In 2015, the same research group investigated BLV infection in 239 breast tissue samples collected from individuals in Alabama, Pennsylvania, Ohio, and California. In 2015, the same research group investigated BLV infection in 239 breast tissue samples. They reported that BLV was detected in 59% of samples from breast cancer patients. In a separate group of patients with premalignant breast changes, the BLV infection rate was 38%. In contrast, the prevalence of BLV infection in non-cancerous breast tissues from healthy individuals was 29%. (Buehring et al., 2015). Analysis of clinical samples provided by the University of Texas revealed that BLV infection, rather than human papillomavirus (HPV), was associated with breast cell carcinogenesis (Baltzell et al., 2018), further supporting a potential link between BLV and breast cancer. The research team also conducted a similar investigation at Columbia University, where detection results similarly indicated an association between BLV infection and breast cell transformation (Olaya-Galán et al., 2021); notably, this study also identified BLV DNA in blood samples from breast cancer patients. Collectively, these findings suggest that BLV infection may be one of the contributing factors in the development of human breast cancer. Importantly, BLV DNA and specific IgG antibodies have been detected in human blood samples (Buehring et al., 2019; Olaya-Galán et al., 2021), indicating that the virus has the potential to disseminate via the bloodstream and access various tissues and organs. The viral load and tissue distribution of BLV require further investigation. It is well established that retroviral infections, such as human T-cell leukemia virus (HTLV), induce cellular carcinogenesis through a prolonged process involving progressive genetic alterations over time. Therefore, the presence of BLV in histologically normal cells suggests a potential for long-term oncogenic progression.

### 4.3 BLV infection investigation in the Australian population

Building on their previous findings, Buehring et al. conducted a follow-up study in Australia. A total of 96 female breast tissue samples were collected and analyzed. The results revealed that bovine leukemia virus (BLV) was detected in 59 (61.5%) of the 96 samples (Buehring et al., 2017). Among these, BLV was identified in 40 (80%) of the 50 samples obtained from breast cancer patients, compared to 19 (41.3%) of the 46 samples from women without a history of breast cancer. Notably, in a subset of patients with available paired tissue samples, BLV was found to be present in benign breast tissue 3 to 10 years prior to the diagnosis of breast cancer in 74% of cases.

These findings further corroborate the results of their earlier investigations in the United States, supporting the hypothesis that BLV infection may play a role in the development of breast cancer.

### 4.4 BLV infection investigation in the Brazilian population

Milk consumption in Brazil is high, and a significant proportion of the population consumes raw milk. Bovine leukemia virus (BLV) infection is regionally prevalent among cattle. Given these factors and existing evidence from prior studies, Brazilian researchers have undertaken investigations into the potential association between BLV infection and human breast cancer. To date, three relevant studies have been published in Brazil. The first study, conducted by the Kreutz et al. research group in southern Brazil in 2019 (Schwingel et al., 2019), included 72 breast cancer tissue samples and 72 healthy breast tissue samples. Nested PCR was used to detect the BLV Tax gene, and partial sequencing of the gag gene was performed for confirmation. The results revealed a BLV positivity rate of 30.5% in breast cancer tissues compared to 13.9% in non-cancerous tissues, suggesting a possible association between BLV infection and the development of human breast cancer. In 2020, Delarmelina et al. reported findings from Minas Gerais state (Delarmelina et al., 2020). This study analyzed 88 breast tissue samples, including 49 from individuals with breast cancer and 39 from those with normal breast tissue. PCR assays targeting both the BLV Tax and Env genes were performed, and amplified products were confirmed by sequencing. BLV DNA was detected in 47 of the 49 breast cancer samples (95.9%) and in 23 of the 39 normal samples (59%), indicating a strong positive association between BLV presence and breast cancer. Notably, a subsequent investigation by Canova et al. (2021) analyzed samples from the same region as Schwingel et al. and found that the BLV Env sequence in human breast tissue shared 97.8–99.7% nucleotide identity with BLV isolates from local bovine blood samples. These genetic similarities suggest that BLV detected in human breast tissues is closely related to viruses circulating in local cattle, supporting the hypothesis of zoonotic transmission.

### 4.5 BLV infection investigation in the Pakistani population

Recently, researchers in Pakistan investigated the potential association between human breast cancer and (BLV) infection. In contrast to previous studies conducted in other countries, Khan et al. collected a large cohort of samples, including 2,710 breast cancer tissue specimens and 80 healthy breast tissue specimens (Khan et al., 2022). PCR was used to detect the BLV Tax and Gag genes to confirm viral infection. The results showed that 728 out of 2,710 cancerous tissue samples were BLV-positive, yielding a positivity rate of 26.8%. Among the 80 healthy tissue samples, 10 were identified as BLV-positive, resulting in an infection rate of 12.5%. These findings suggest a significant association between BLV infection and human breast cancer. Furthermore, the authors recommend enhanced monitoring of local cattle populations and dairy products to help mitigate the transmission of BLV.

### 4.6 BLV infection investigation in the Iranian population

Similar to previous reports, researchers in Iran conducted an investigation into the potential association between BLV infection and breast cancer within their national population. In this study, PCR was employed to detect the BLV Tax and Gag genes as markers of viral infection. Khalilian et al. analyzed 200 tumor tissue samples, including 172 confirmed cancer cases and 28 suspected cases, along with 200 blood samples from healthy individuals (Khalilian et al., 2019). Their results showed that 30% of tumor samples were BLV-positive, compared to 16.5% of samples from healthy individuals. These findings indicate a significant association between BLV infection and the occurrence of breast cancer.

### 4.7 BLV infection investigation in the Chinese population

Scholars at Yangzhou University in China conducted a study to investigate the potential association between bovine leukemia virus (BLV) infection and breast cancer. Samples were collected from Anhui, Jiangsu, and Shanghai in 2016 (Zhang et al., 2016), including 91 breast cancer tissue samples, 160 blood samples from breast cancer patients, and 100 blood samples from non-breast cancer individuals.

Concurrently, 150 bovine blood samples were collected from cattle in Anhui, Beijing, Jiangsu, and Heilongjiang provinces. All samples underwent nucleic acid and serological testing using PCR, real-time PCR, and ELISA methods. The results showed that the BLV positive rate in bovine blood samples was approximately 50%, whereas neither BLV nucleic acid nor antibodies were detected in human breast cancer tissues or blood samples, indicating no evidence of an association between BLV infection and breast cancer in this cohort.

### 4.8 BLV infection investigation in the Japanese population

Recently, Yamanaka et al. conducted a preliminary investigation into BLV infection among the Japanese population (Yamanaka et al., 2022). In this study, 97 human blood samples and 23 tissue samples were analyzed for the presence of BLV. Using PCR, no BLV nucleic acid was detected; similarly, ELISA failed to detect anti-BLV IgG or IgM antibodies. These results indicate the absence of BLV infection in the tested samples. Milk consumption in Japan is relatively high; however, the intake of raw milk is low, which may explain the lack of BLV detection in this population.

### 4.9 BLV infection investigation in the Egyptian population

Recently, a study conducted by an Egyptian research group utilizing real-time PCR assays reported that the association between BLV and breast cancer progression remains statistically inconclusive (Raouf et al., 2025), yet reveals notable clinical trends. BLV DNA was detected in 22.7% of breast cancer tissue samples compared to 16% in control tissues; however, this difference did not reach statistical significance. Furthermore, no significant correlation was observed between isolated BLV infection and key clinicopathological parameters, including tumor size, histological grade, or hormone receptor status. Nonetheless, several findings indicate a potential association with more aggressive disease features. BLV-positive cases demonstrated higher frequencies of lymphovascular invasion (70.6% vs. 53.4%) and advanced-stage disease (Stage III: 35.3% vs. 22.4%) relative to BLV-negative cases, although these differences remained below the threshold for statistical significance in this cohort. Notably, the co-detection of multiple viruses—particularly BLV and Epstein-Barr virus (EBV)—was significantly associated with younger patients diagnosed with breast cancer, suggesting a possible synergistic effect of viral co-infection in earlyonset or more aggressive forms of the disease.

In addition, in a separate study, Gillet et al. investigated the potential association between BLV infection and breast cancer by analyzing whole-genome sequencing data from 51 breast cancer cases (Gillet and Willems, 2016). No BLV nucleic acid sequences were identified, suggesting that these tumors were not associated with BLV infection.

**Figure 2:**
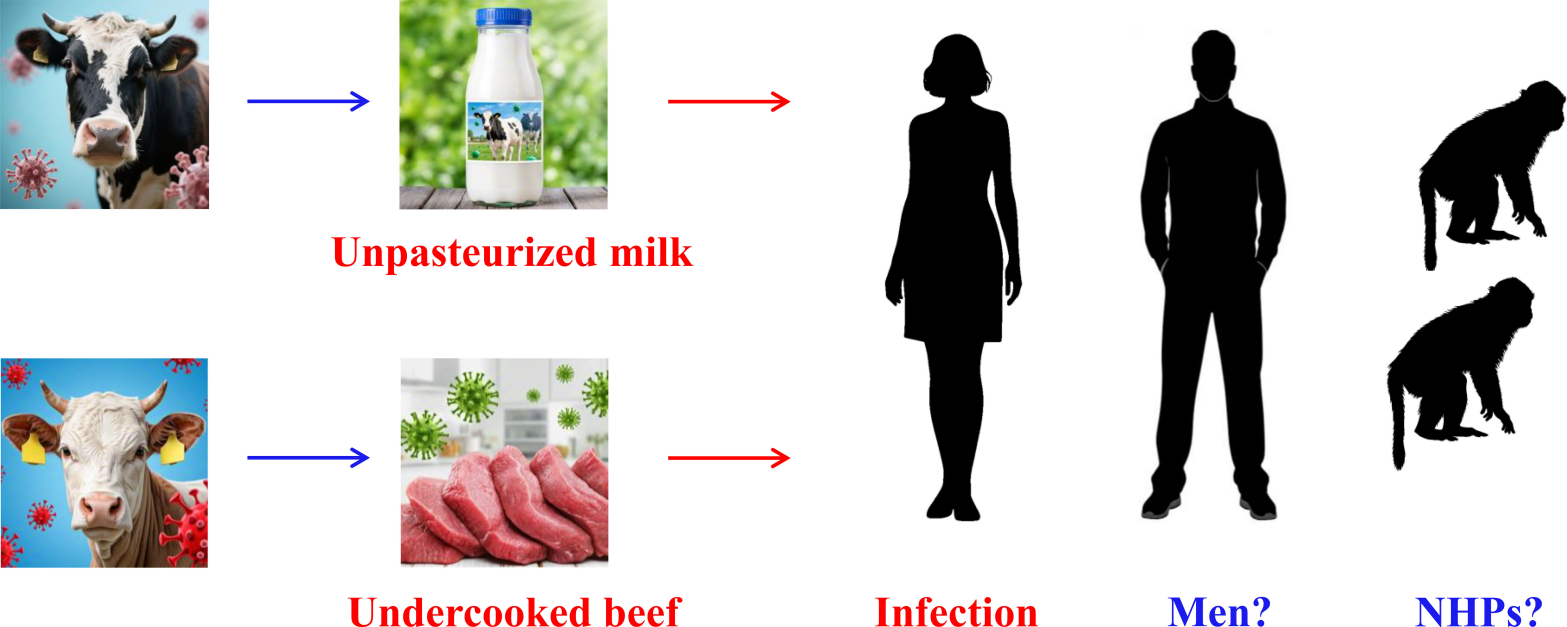
Schematic representation of transmission routes from BLV raw products to humans. Currently, there are no published reports documenting BLV infection in males, and experimental evidence of BLV infection in non-human primates (NHPs) remains lacking.

Based on the available reports, researchers across multiple countries have conducted studies on the possible link between BLV infection and breast cancer, yet the findings remain inconsistent. It has been demonstrated that BLV can establish stable infection in human cells (Olaya-Galán et al., 2022). Logically, a potential epidemiological association between BLV and breast cancer would require two key conditions: (1) endemic BLV infection in regional cattle populations, and (2) local dietary habits involving consumption of raw milk or undercooked beef (**Figure 2**). The dietary patterns in the United States, Brazil, and Pakistan align with these conditions. In contrast, current reports from China and Japan have not detected BLV in breast tissue samples, which may be attributed to limited consumption of raw dairy products in these countries. Nevertheless, further in-depth investigations are needed to fully understand the prevalence and transmission dynamics of BLV in such populations. Future studies should expand surveillance to regions where both risk factors are present. Notably, existing research has primarily focused on female populations; the status of BLV infection in males remains unclear and warrants evaluation in future clinical studies (**Figure 2**). Importantly, strategies for preventing and eliminating BLV infection in both cattle and humans represent critical public health challenges that require urgent attention.

## 5 BLV vaccination and control

Vaccines play a critical role in preventing pathogenic infections. BLV is a member of the retrovirus family and exhibits low replication fidelity, which poses significant challenges for the development of preventive vaccines. Retroviral vaccine development is widely recognized as a major scientific hurdle. For example, despite substantial investment of human and financial resources, an effective vaccine against human immunodeficiency virus (HIV/AIDS) remains elusive (Barouch, 2008). To date, the most successful retroviral vaccine is the attenuated equine infectious anemia virus (EIAV) vaccine, developed by Rong-Xian Shen (Harbin Veterinary Research Institute, Chinese Academy of Agricultural Sciences) in the 1980s (Lin et al., 2011). This vaccine was generated through serial passage of a highly virulent strain, enabling horses to develop protective immunity against subsequent challenge with wild-type virus. The success of the EIAV vaccine has provided valuable insights for the development of HIV and other retroviral vaccines. In Fact, extensive efforts were made toward BLV vaccine development during the 1980s in China. Attempts to apply similar attenuation strategies used for EIAV proved ineffective, as virulent BLV strains were difficult to attenuate through conventional methods, making it challenging to obtain a stable live-attenuated strain. Alternative approaches using traditional whole-virus inactivated or subunit vaccines were also explored but failed to confer robust protection against BLV infection.

Recently, a research team reported the development of a genetically modified BLV vaccine (developed by the research group of Professor Luc Willems). This candidate vaccine strain was created by deleting specific regions of the BLV genome—R3, G4, AS1-S, and AS1-L—along with introducing mutations in the Env gene, resulting in viral attenuation (Suárez Archilla et al., 2022). Following immunization of cattle with this strain, the vaccine virus exhibited slow replication kinetics and was capable of eliciting host humoral immune responses. The strain demonstrated genetic stability and showed no evidence of vertical transmission from vaccinated cows to their calves. Cattle immunized with this attenuated vaccine were protected against challenge with virulent BLV strains for up to four years, indicating its potential for controlling BLV spread. Nevertheless, further evaluation is required to determine whether this vaccine can induce sufficient herd immunity and provide consistent protection across diverse field conditions. In addition, novel vaccine technologies may offer a potential alternative for achieving sterilizing immunity against BLV infection (Tang et al., 2024).

As discussed above, numerous studies have reported an association between BLV infection and human breast cancer (Saeedi-Moghaddam et al., 2024; Blanco et al., 2025); however, these findings remain largely observational and require deeper, systematic validation. It is noteworthy that the majority of studies reviewed in this article, including those conducted in the United States and Brazil (Buehring et al., 2014; Canova et al., 2021), have indeed analyzed human-derived bovine leukemia virus (BLV) sequences and performed preliminary comparisons with prevalent strains circulating in local cattle populations. Nevertheless, current evidence remains limited by factors such as uneven geographical distribution and small sample sizes, which preclude definitive conclusions regarding the transmission of BLV from local cattle to humans. To conclusively establish transmission, highly homologous viral sequences from both local bovine and human sources should be systematically documented across a broader geographic range.

Buehring et al. co-cultured FLK cells, which produce endogenous BLV with human cells, then isolated the infected human cells and maintained them in culture for 3–6 months. These experiments demonstrated that human cells could sustain a stable BLV infection over time (Olaya-Galán et al., 2022), providing strong evidence that BLV can infect human cells and establish persistent infection. This supports the possibility of BLV infection in human breast tissue. However, if BLV is implicated in breast carcinogenesis, the molecular mechanisms by which it may contribute to the transformation of mammary epithelial cells would represent a critical area of scientific investigation. As a retrovirus, BLV has the theoretical capacity to integrate its proviral DNA into various regions of the host genome. Should integration occur in proximity to cellular proto-oncogenes through mechanisms such as promoter insertion, enhancer activation, or genomic disruption, it may trigger their aberrant expression and thereby contribute to malignant transformation. Consequently, whole-genome sequencing of BLV-positive clinical breast tumor samples is recommended to accurately identify and map viral integration sites within the host genome. Such an approach would provide critical insights into the potential role of BLV in mammary carcinogenesis via proto-oncogene activation.

Non-human primates (NHPs), being the closest animal models to humans (Yu et al., 2013), should offer a promising system for studying BLV pathogenesis. For instance, NHPs could be orally exposed to raw milk or food contaminated with BLV (**Figure 2**). Subsequently, researchers could monitor whether BLV establishes productive infection, assess serum antibody levels, and quantify viral load in various tissues, particularly breast tissue. Such studies would help elucidate the *in vivo* dynamics of BLV infection in a physiologically relevant model and provide critical insights into potential routes of human infection.

If a causal relationship between BLV infection and human breast cancer genesis is confirmed, strategies for eliminating BLV from infected individuals (both human and bovine) will become essential. As illustrated above, given the difficulties in developing effective vaccines, alternative approaches are warranted. The CRISPRCas9 gene-editing technology (clustered regularly interspaced short palindromic repeats-associated protein 9) has emerged as a powerful tool for therapeutic applications (Madigan et al., 2023). Recently, CRISPR-Cas9-based therapy received regulatory approval in the UK for the treatment of sickle cell disease in individuals aged 12 years and older and transfusion-dependent β-thalassemia. These advances highlight the clinical feasibility of gene editing. Therefore, it is worth exploring the use of CRISPR-Cas9 to target and eliminate integrated or latent BLV proviral DNA in infected cells. For example, building upon the research methodology of Wang et al (Wang et al., 2020), an adenovirus-based CRISPR-Cas9 system could be designed to target BLV. This system utilizes a guide RNA (gRNA) specifically engineered to recognize and cleave integrated BLV proviral DNA within the host genome, thereby enabling its elimination. In addition, with the advancement of modern artificial intelligence, bioinformatics is expected to play an increasingly significant role in the design and development of vaccine (Yu et al., 2024). Such approaches may offer a novel strategy for developing prophylactic or therapeutic interventions aimed at eradicating persistent BLV infections in both cattle and humans.

## 6 Conclusion

BLV is widely prevalent across many countries and regions and is difficult to eradicate in the short term. As a retrovirus, BLV poses significant challenges for vaccine development. Therefore, routine monitoring, along with the prompt isolation and culling of infected cattle, remains an effective strategy for controlling BLV transmission. In recent years, multiple studies from various countries have reported an association between BLV infection and human breast cancer development, suggesting the potential for cross-species transmission. Enhanced surveillance of dairy products in BLV-endemic areas, coupled with modifications to dietary habits, particularly the avoidance of raw milk consumption, could help reduce human exposure to BLV and may contribute to lowering the incidence of BLV-associated breast cancer. Meanwhile, the successful development of an effective BLV vaccine would play a crucial role in long-term control and eventual eradication of the virus in both animal and human populations.
